# A prospective study of absolute risk and determinants of human papillomavirus incidence among young women in Costa Rica

**DOI:** 10.1186/1471-2334-13-308

**Published:** 2013-07-08

**Authors:** Megan Clarke, Mark Schiffman, Sholom Wacholder, Ana Cecilia Rodriguez, Allan Hildesheim, Wim Quint

**Affiliations:** 1 Division of Cancer Epidemiology and Genetics, National Cancer Institute, National Institutes of Health, DHHS, Bethesda, MD, USA; 2 Proyecto Epidemiológico Guanacaste, San José, Costa Rica; 3 Delft Diagnostic Laboratories, Rijswijk, Netherlands

**Keywords:** Human papillomavirus, Absolute risk, Risk factors, Sexual behavior

## Abstract

**Background:**

High risk human papillomaviruses (HR-HPV) are known to be extremely common, sexually transmitted infections, but more information is needed regarding the absolute risks of type-specific HR-HPV infections in the years following sexual debut.

**Methods:**

We conducted a survival analysis of 3,737 women aged 18–25 from the control group of the Costa Rican Vaccine trial to determine the absolute risks of HR-HPV infections at 12 months, 24 months, and end of follow-up (average of 50.7 months). To corroborate determinants of infection, we used Cox proportional hazards methods to assess associations between demographics and sexual risk behaviors and incident HR-HPV.

**Results:**

Cumulative incidence for HR-HPV infections was 51.3% at the end of the study period. The most common incident types were HPV52 (15.4%), HPV51 (13.6%), and HPV16 (12.4%). Type-specific cumulative incidence corresponded closely with type-specific prevalences, except that HPV16 was more prevalent than predicted by incidence, suggesting greater persistence. The strongest predictors of incident HR-HPV infections as a group in a multivariate analysis were the expected correlates of sexual behavior of the woman and her partner, such as being single (HR 1.6, 95% CI 1.4-1.8) or divorced/widowed (HR: 2.1, 95% CI: 1.7-2.7), having multiple HPV infections at enrollment (HR: 1.5, 95% CI: 1.3-1.7), and current smoking (HR: 1.2, 95% CI: 1.0-1.3). In women who reported being having only one lifetime sexual partner (being in a monogamous relationship), the strongest predictors of HR-HPV included not living with sex partner (HR: 2.1, 95% CI 1.7-2.5) and age of sex partner (HR: 1.4, 95% CI: 1.0-1.8).

**Conclusion:**

We confirm the extremely high incidence of HR-HPV in young women, emphasizing the importance of vaccinating young girls before sexual debut.

## Background

Human papillomavirus (HPV) infections are easily transmitted by sexual contact and are very common among sexually active young women [1,2]. Approximately 90% of all HPV infections will clear (or become undetectable using standard methods) within a few years, yet a small proportion of persistent, carcinogenic HPV infections (HR-HPV) will eventually cause cervical cancer if precancerous lesions are not treated [3]. Currently, a prophylactic vaccine is available for HR-HPV types 16 and 18. The vaccine efficacy is high for types 16 and 18, and has also been shown to provide partial protection for types 31, 33, and 45 [4], yet other HR-HPV types are not known to be at all protected against by the vaccine. The absolute risks of acquiring individual cervical HR-HPV infection are not adequately defined; such analyses are surprisingly uncommon in the literature because they require large-scale HPV typing of sizable longitudinal studies.

The determinants of HPV infection acquisition, all types combined, are better defined. Risk for HPV acquisition is strongly associated with sexual behavior [1,3,5]. There is evidence to support that use of condoms and circumcision of the male partner reduces rates of HPV transmission [6,7].

Characterizing more fully the incidence of new HR-HPV infections in young sexually active women would be informative for models of HR-HPV transmission and vaccination impact. The Costa Rican Vaccine Trial (CVT) control arm provides an opportunity to study a large number of recently acquired HR-HPV infections within a group of young women aged 18–25 years old. Such a young and narrow age range enables us to look at the acquisition of new HR-HPV infections, presumably around the onset of sexual activity. The primary purpose of this study was to define the absolute risk of HR-HPV and type-specific HR-HPV infection in our cohort of young women. Secondarily, we wished to add to the already substantial literature on determinants of transmission. We were also interested in confirming or refuting our prior belief that different HPV types (e.g., high-risk vs. low-risk) have similar predictors.

## Methods

### Study participants

The study population consisted of all 3,739 women participating in the control group of the Costa Rican Vaccine Trial (CVT; NCT00128661)). Study design and procedures have been described elsewhere [8]. Briefly, CVT is a community-based, double-blind randomized phase III trial aimed at evaluating the efficacy of HPV 16/18 bivalent vaccine in preventing cervical precancers. At enrollment, women provided written informed consent. The trial recruited 7,466 women aged 18–25 years from the Guanacaste and Puntarenas provinces, Costa Rica. The vaccination schedule for both groups was three doses: one at enrollment, one month, and six months later. After receiving the vaccines, women were followed once per year for at least four years if their cytology was normal, and were transferred to a six-month follow-up schedule if they had HPV-related cytological abnormalities. We excluded women in the vaccine arm because we wanted to measure the true incidence and determinants of HR-HPV acquisition in an unvaccinated population.

All participants were administered an enrollment questionnaire by a trained interviewer. The questionnaire elicited information on level of education, marital status, household facilities, menstrual history, sexual, reproductive, and contraceptive history, smoking, and family history of cancer. Among women who reported being in a monogamous relationship, additional questions were asked about their sexual partner including their age, education, circumcision status, sexual history, and smoking history. All study protocols were reviewed and approved by the National Cancer Institute and Costa Rican Institutional Review Boards.

### HPV DNA testing

Exfoliated cervical cells were collected using a Cervex brush and rinsed into a vial of 20 mL of PreservCyt solution during the pelvic exam. Samples were tested for HPV DNA by polymerase chain reaction (PCR) amplification with SPF_10_ primers followed by DNA enzyme immunoassay detection of amplimers. HPV typing on positive amplimers was performed using line probe assay (LiPA_25_). All HPV positive samples that were HPV16/18 negative by LiPA_25_ were tested by type-specific PCR. Carcinogenic HPV types, termed high-risk HPV (HR-HPV) in this analysis, included 16,18,31,33,35,39,45,51,52,56,58, and 59 . Other HPV types, characterized as low-risk HPV (LR-HPV) included 6, 11, 34, 40, 42, 43, 44, 53, 54, 66, 68, 70, 73, 74, and unknown types. The typing assay does not distinguish HPV68 and HPV73: they were included conservatively as low-risk.

### Statistical analysis

For each individual HPV type, we estimated the 12 and 24 month and overall cumulative incidence. Incidence implied new detection of that HPV type for women who were or became sexually active who were completely HPV negative at enrollment or who were positive only for other types. Months at risk were calculated on the basis of number of months from the enrollment visit to detection of an incident HPV type or at the last follow-up visit, if a woman remained negative for that type throughout the study. Event times were calculated as the midpoint of the intervals between the last HPV type negative and the first HPV type positive visit.

Socio-demographic, sexual behavior, contraceptives, smoking and reproductive history characteristics measured at enrollment, as well as prevalent HPV infection and number of prevalent HPV types were evaluated as possible determinants of incident HR-HPV and LR-HPV infection. Continuous questionnaire variables were categorized based on trends in the data when appropriate. The five questions regarding household facilities were characterized as an overall measure of socioeconomic status (SES). Women who had all five household amenities (electricity, refrigerator, toilet, television, and running water) were considered to have high SES while women who had less than all five were categorized as low SES. The variable, years of sexual activity was calculated by subtracting the women’s age at sexual debut from her age at enrollment. Questionnaire variables that only pertained to women having only one lifetime sexual partner were evaluated separately. From now on these women will be referred as being in “monogamous” relationships.

Cox proportional hazards regression models were used to evaluate the relationship between enrollment questionnaire variables and time to incident HPV infection. Variables that were significant at the p ≤ 0.10 level or below in the univariate analysis as well as those that were of particular interest for this study, (i.e. circumcision status and condom use) were considered in multivariate regression models. The final models included variables that were significant at the p ≤ 0.05 threshold and collinearity between covariates was assessed by calculating variance inflation factors for each variable after fitting the mulitvariable regression models. Two women were excluded for having a questionnaire that was deemed unreliable by the interviewer, leaving a total of 3,737 women in the final analysis. All analyses were conducted using STATA (version 11.0; StataCorp).

## Results

### Prevalent and incident HR-HPV infections

**Figure 1:**
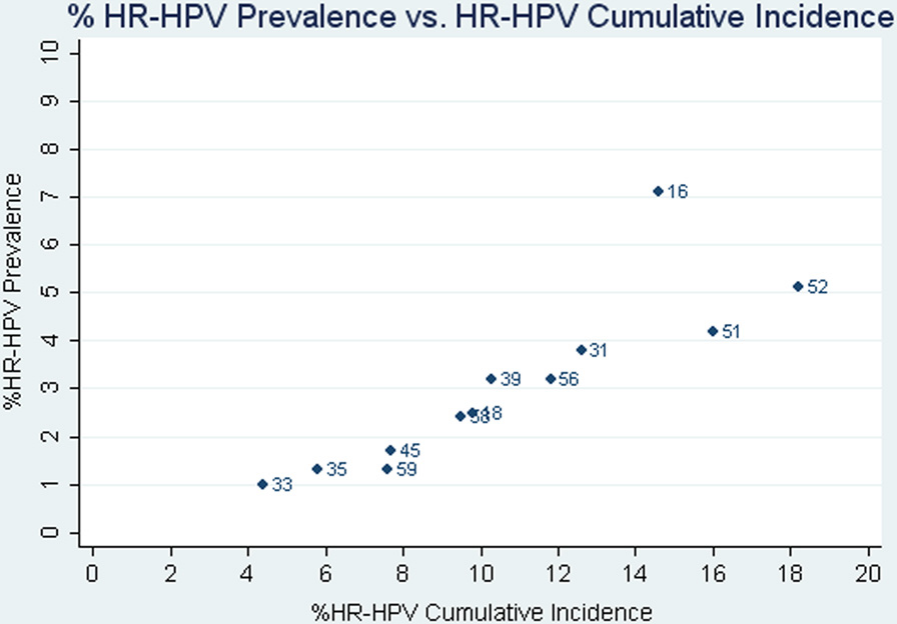
A plot of the percent prevalence versus percent cumulative incidence by HR-HPV type.

On average, women attended 4.476 screening follow-up visits throughout the study. Total person-months at risk was estimated to be 114,062.8 months for all HR-HPV types (data not shown). Table 1 shows the type-specific prevalence and cumulative incidence of HR-HPV, LR-HPV, and type-specific HR-HPV by time intervals, ending at the end of follow-up (averaging about 50 months for most women). HPV typing data was available for 3,731 women. At enrollment, 947 (25.3%) women were HR-HPV positive and 812 (21.8%) were LR-HPV positive. HPV16 was the most common prevalent type (7.1%), followed by HPV52 and HPV51, respectively. Cumulative incidence was slightly higher for LR-HPV types compared to HR-HPV types (54.3% vs. 51.3%, respectively). Type-specific HR-HPV cumulative incidence varied, with HPV52 being the most common (15.4%, 95% CI: 14.3-16.7%), followed by HPV51 (13.6%, 12.5-14.7%) and HPV16 (12.4%, 11.4-13.5%). Figure 1 shows a plot of type-specific HR-HPV prevalence versus cumulative incidence. A correlation was observed between the most common prevalent and incident types, with the exception of HPV16, which was highly prevalent compared to all other HR-HPV types, but had the third highest cumulative incidence. The cumulative incidence of multiple HR-HPV types was 32.9% (31.3-34.7%) and a total of 1,022 women remained HPV negative throughout the entire study (data not shown).

**Table 1:** Prevalence and cumulative incidence of HR-HPV, LR-HPV, and HR-HPV types among 3,731 women in the Costa Rican Vaccine Trial

	**% HPV positive at enrollment**	**% HPV negative at enrollment**	**% Cumulative HPV incidence**^**# **^**12 months**	**% Cumulative HPV incidence 24 months**	**% Cumulative HPV incidence end of follow-up**^**^**^
HR-HPV	25.3	74.7	29.4	43.0	51.3
LR-HPV	21.8	78.2	31.7	47.4	54.3
HPV16	7.1	92.9	5.7	9.8	12.4
HPV18	2.5	97.5	3.5	6.3	8.2
HPV31	3.8	96.2	4.7	8.0	10.5
HPV33	1.0	99.0	1.7	3.1	3.8
HPV35	1.3	98.7	2.4	3.6	4.8
HPV39	3.2	96.8	3.7	6.1	8.3
HPV45	1.7	98.3	3.0	5.3	6.5
HPV51	4.2	95.8	6.1	10.8	13.6
HPV52	5.1	94.9	6.3	12.1	15.4
HPV56	3.2	96.8	4.0	7.5	9.8
HPV58	2.4	97.6	3.6	6.0	7.8
HPV59	1.3	98.7	2.4	4.9	6.3

# Incidence defined by new detection of HPV type for women who were completely HPV negative at enrollment or who were positive only for other types.

^Average end of follow-up is 50.7 months, very few women had a maximum end of follow-up of 76 months.

HR, High Risk; LR, Low Risk.

### Univariate associations for risk of incident HR-HPV

Table 2 presents the univariate associations for HR-HPV incidence among the 3,737 women included in this study. Within the restricted age range, age at enrollment was not related to HR-HPV acquisition. HR-HPV acquisition was significantly associated with being single or divorced/widowed, high SES, not being a virgin at enrollment, not being in a monogamous relationship, fewer years since initiation of sexual activity, greater number of sex partners, and a history of cigarette smoking within the past six months. Lifetime OC use and lifetime injectable contraceptive use were protective against incident HR-HPV while history of condom use and use of barrier methods (both condoms and diaphragms) significantly increased the risk of HR-HPV acquisition. Being positive for any HPV type at enrollment significantly increased the risk of incident HR-HPV infection, as did being infected with more than one HPV type. We assessed whether history of condom use was confounded by increased frequency of sexual activity and found that risk of HR-HPV was no longer associated with condom use when adjusting for number of sexual partners. To determine whether being single or divorced was a proxy for more sexual relationships, we stratified the analysis of marital status by whether a woman was in a monogamous relationship. However, the hazard ratios (HR) were similar for women who were not in a monogamous relationship (single: HR: 1.1, 95% CI: 1.0-1.3 and divorced: HR: 2.0, 95% CI: 1.5-2.6) and women who were in a monogamous relationship (single: HR: 1.9, 95% CI: 1.6-2.1 and divorced: HR 2.0, 95% CI: 1.5-2.7). We observed very similar univariate associations for LR-HPV types (data not shown).

**Table 2:** Univariate cox proportional hazards regression analysis for incident HR-HPV among Women in the Costa Rican Vaccine Trial

**Woman characteristics**		**Total**		**Positive for one or more new HR-HPV types**		
		**n**	**Col %**	**n**	**%**	**HR (95% CI)**
Age at Enrollment (years)	18-20	1,729	46.4	864	50.0	1.1 (1.0 – 1.2)
	21-25	2,002	53.6	947	47.3	Ref
Marital Status	Divorced/Widowed	98	2.6	70	71.4	2.5 (1.9 – 3.1)
	Single	2,082	56.0	1,084	52.1	1.4 (1.3 – 1.5)
	Married or living as	1,544	41.4	654	42.4	Ref
Measures of SES	All 5	3,012	80.7	1,517	50.4	1.3 (1.2 – 1.5)
	<5	719	19.3	294	40.9	Ref
Virgin at Enrollment	No	2,918	78.2	1,543	52.9	2.0 (1.7 – 2.2)
	Yes	813	21.8	268	33.0	Ref
Years Sexually Active	<1-2	674	23.1	393	58.3	1.4 (1.2 – 1.7)
	3-4	812	27.9	430	53.0	1.2 (1.1 – 1.5)
	5-7	890	30.6	461	51.8	1.1 (1.0 – 1.3)
	>7	536	18.4	256	47.8	Ref
Monogamous Relationship	No	2,489	66.7	1,253	50.3	1.2 (1.1 -1.4)
	Yes	1,242	33.3	558	44.9	Ref
Number of Sex Partners	3+	902	31.2	560	62.1	1.7 (1.5 – 1.9)
	2	749	25.9	408	54.5	1.4 (1.2 – 1.6)
	1	1,242	42.9	558	44.9	Ref
Duration Sexual Relationship with Partner (days)	1-330	1,051	36.3	608	57.9	1.5 (1.3 – 1.7)
	331-1200	1,022	35.3	546	53.4	1.3 (1.2 – 1.5)
	1201-4380	824	28.4	376	45.6	Ref
Lifetime Oral Contraceptive Use	No	562	20.0	360	64.1	1.5 (1.3 – 1.7)
	Yes	2,255	80.0	1,124	49.8	Ref
Lifetime Injectable Contraceptive Use	No	1,685	59.8	911	54.1	1.2 (1.0 – 1.3)
	Yes	1,133	40.2	571	50.4	Ref
Lifetime Condom Use	Yes	1,729	61.3	933	54.0	1.2 (1.1 – 1.3)
	No	1,092	38.7	550	50.4	Ref
Use of Barrier Methods During Sex	Condom/Diaphragm	788	27.1	465	59.0	1.3 (1.2 – 1.4)
	Neither	2,123	72.9	1,074	50.6	Ref
Smoked Cigarettes in Past 6 Months	Yes	499	13.4	317	63.5	1.7 (1.5 – 1.9)
	No	3,225	86.6	1,491	46.2	Ref
HPV Positive at Enrollment	Yes	1,459	39.1	902	61.8	2.0 (1.8 – 2.2)
	No	2,272	60.9	909	40.0	Ref
Number of Enrollment HPV Infections	2 or more types	549	14.7	361	65.8	2.3 (2.0 – 2.6)
	1 type	910	24.4	541	59.5	1.9 (1.7 – 2.1)
	0 types	2,272	60.9	909	40.0	Ref

Among women who were in monogamous relationships (Table 3), younger age of sex partner, sex partner having had sexual intercourse with more than one other woman in their lifetime, not living with sex partner, and sex partner having a lifetime history of smoking were significantly associated with increased risk of incident HR-HPV infection. Male partner’s circumcision status (reported by female partner) was not correlated with a decreased risk of HR-HPV acquisition, although the response rate for this question was very low (about 1/3 of participants). Similar associations were observed for LR-HPV types (data not shown).

**Table 3:** Univariate cox proportional hazards regression analysis for incident HR-HPV among women in monogamous relationships in the Costa Rican Vaccine Trial

		**Total**		**Positive for one or more new HR-HPV types**		
**Woman characteristics**		**N**	**Col (%)**	**N**	**%**	**HR (95% CI)**
Age of Sex Partner (years)	16-24	542	43.8	268	49.5	1.4 (1.1 – 1.8)
	25-30	483	39.1	207	42.9	1.1 (0.9 – 1.5)
	>30	212	17.1	80	37.7	Ref
Number of Other Women Sex Partner has had Sex with	2+	467	47.6	228	48.8	1.2 (1.0 – 1.6)
	1	271	27.6	109	40.2	1.0 (0.7 – 1.3)
	0	243	24.8	104	42.8	Ref
Lives with Sex Partner	No	458	36.9	270	59.0	2.1 (1.8 – 2.5)
	Yes	784	63.1	288	36.7	Ref
Partner is Circumcised	Yes	604	52.9	267	44.2	1.0 (0.9 – 1.2)
	No	538	47.1	241	44.8	Ref
Sex Partner Ever Smoke	Yes	355	28.6	188	53.0	1.3 (1.1 – 1.6)
	No	886	71.4	370	41.8	Ref

### Multivariate analysis for risk of incident HR-HPV

In the multivariate model shown in Table 4, after controlling for age at enrollment, significant associations for acquisition of a new HR-HPV type included being single or divorced/widowed, high SES, shortest time since initiation of sexual activity (<1-2 years), not being in a monogamous relationship, never using birth control pills, history of cigarette smoking within the past six months, and being positive for one or more HPV infections at enrollment. For LR-HPV types, only marital status and number of enrollment HPV infections were significant in the multivariate model (data not shown). Among women in monogamous relationships, not living with your sex partner, young age of sex partner, partner’s lifetime history of smoking, and sex partner having intercourse with two or more other women were significantly associated with increased risk of incident HR-HPV. Similar associations were observed for LR-HPV types, with the exception of age of sex partner which was not included in the model (data not shown).

**Table 4:** Multivariate cox proportional hazards regression analysis for risk of incident HR-HPV among all women and women in monogamous relationships in the Costa Rican Vaccine Trial

**All women**		
**Women characteristics**		**HR (95% CI)**
Marital Status	Married	Ref
	Single	1.7 (1.4 – 1.8)
	Divorced/Widowed	2.1 (1.7 – 2.7)
Measures of SES	Less than 5	Ref
	All 5	1.2 (1.0 – 1.4)
Years Sexually Active	0-2	1.3 (1.1 – 1.6)
	3-4	1.2 (1.0 – 1.4)
	5-7	1.1 (1.0 – 1.3)
	>7	Ref
Monogamous Relationship	Yes	Ref
	No	1.4 (1.2 – 1.6)
Lifetime Oral Contraceptive Use	Yes	Ref
	No	1.2 (1.0 – 1.3)
Smoked Cigarettes Past 6 Months	No	Ref
	Yes	1.2 (1.0 – 1.4)
Number of Enrollment HPV Types	0	Ref
	1	1.4 (1.3 – 1.6)
	2+	1.5 (1.3 – 1.7)
**Women in Monogamous Relationships Only**		
Lives with Sex Partner	Yes	Ref
	No	2.1 (1.7 – 2.5)
Age of Sex Partner (years)	16-24	1.4 (1.0 – 1.9)
	25-30	1.4 (1.0 – 1.8)
	>30	Ref
Sex Partner Ever Smoke	No	Ref
	Yes	1.3 (1.1 – 1.6)
Number of Other Women Sex Partner has had Sex with	0	Ref
	1	1.0 (0.8 – 1.3)
	2+	1.3 (1.0 – 1.6)

### Risks for the three most prevalent and incident HR-HPV types: 16, 51, and 52

To determine if infection with the three most prevalent HR-HPV types influenced the subsequent incidence of other HR-HPV types, we conducted a Cox proportional hazard analysis in which prevalent infection with HPV16, HPV52, or HPV51 was used as a predictor of incident infection with other HR-HPV types. As predicted, we found that risk of incident HR-HPV infection of any type significantly increased if a woman was positive for HPV16, HPV52, or HPV51 at enrollment (data not shown).

In the univariate analysis of risk factors for HR-HPV incidence, we observed similar associations for HPV16, HPV51, and HPV52 to all HR-HPV types.

## Discussion

Our study confirms the high incidence of HR-HPV among sexually active young women. HPV16 was the most common prevalent type at study enrollment and HPV52 was the most frequently detected incident type, followed by HPV51 and HPV16. A graphical representation of prevalent and incident HPV types confirms a correlation between frequency of prevalent types and incident types, with the exception of HPV16. The high prevalence of HPV16 relative to its incidence might suggest longer persistence. As expected, risk of incident HR-HPV infection was strongly influenced by variables related to sexual behavior. Both HR-HPV and LR-HPV had similar risk predictors for incident infection.

The high incidence rates we observed in our study are expected from a population of young sexually active women and are similar to other prospective studies of HPV incidence among young women in the United States, Denmark, Colombia, Brazil, and Canada [3,9-12]. Many of the risk factors for incident HR-HPV observed in this study have been reported elsewhere and confirm that risk of incident HR-HPV infection is strongly influenced by sexual behavior [1,3,9,13]. High SES was associated with increased risk of HR-HPV. Some population-based surveys have reported that higher SES might sometimes be associated with a greater number of sexual partners, at least among males [14], though there are no data in the literature to suggest that high SES increases risk for HPV infection apart from a correlation with sexual behavior. The association between recent smoking history and partner's smoking history is also not well understood. In addition to our current findings, other studies that have shown a positive relationship between risk of HPV infection and smoking, suggesting it may be a proxy for high risk sexual behavior [10,15,16]. There is some, albeit inconsistent, evidence suggesting that smoking may influence the immune system [17]. Studies assessing the risk of incident HPV infection and OC use have also been inconsistent. Our findings indicate that lifetime OC use has a protective effect against HR-HPV acquisition and is in line with similar studies [13,18], yet others report no association between OC use and incident HPV infection or even increased risk [19-22].

We did not find evidence suggesting that condom use was protective against HR-HPV acquisition, if anything our data shows an increased risk of HR-HPV infection with condom use, although this association was rendered insignificant when controlled for other measures of sexual behavior. There is conflicting data in the literature, several cross-sectional studies have shown that condom use by male partners does not reduce the risk of HPV infection in women [10], yet others suggest that consistent use of male condoms do effectively reduce the risk of HPV transmission [6]. Since in our study, condom use was a proxy for sexual activity, the modest association between increased HR-HPV and reported condom use might be explained by the fact that condoms are often not worn during every sexual act and even when worn, there could still be transmission.

We found no association between circumcision of male partners and reduced HR-HPV risk among women in monogamous relationships. The lack of association in our study could be due to reliance on the woman’s report of her male partner’s circumcision status, which may be unreliable. Furthermore, only one-third of the women included in this study responded to this this question, therefore the estimation of male partner’s circumcision stats may have been inflated. Previous studies have shown that male circumcision is protective against HPV transmission from male to female partners [23,24]. However, a recent meta-analysis showed no association between male circumcision and HPV incidence among several pooled studies [25].

Limitations in our study included the fact that we were only able to use baseline questionnaire data and therefore were not able to evaluate associations between time-dependent variables and risk of incident HR-HPV infection. Specifically, we did not have any follow-up information on a woman’s sexual activity. Therefore, we do not know if women who reported being a virgin at enrollment, later became sexually active during the course of the study. This may bias our estimates of incidence, because we cannot determine whether these women are truly at risk. However, HPV is also transmissible through modes other than nonpenetrative sexual contact, and HPV has been detected among virgins in other studies [26,27]. The questionnaire was administered as an in-person interview during which participants may have been reluctant to disclose personal information regarding their sexual history resulting in potential response bias. For example, our assessment of condom use included broad categories such as "always" or "never" and was most likely an overestimation of condom use among women of reproductive age in Costa Rica [28]. Our estimation of male circumcision status was based on the woman's recall and a large proportion of women did not respond to this question, therefore this variable was highly subject to bias and misclassification, and probably reflects an overestimation of circumcision among Costa Rican men [29]. Our study had several strengths including the large number of women in our cohort and the narrow age range which allowed us to observe the early natural history of incident HPV infections among sexually active young women. The availability of HPV typing data allowed us to measure the cumulative risk for type-specific HR-HPV infections.

## Conclusions

Our study provides good estimates of HR-HPV, LR-HPV, and type specific HR-HPV infections among a large cohort of sexually active young women. We have confirmed the sexually transmissible nature of HR-HPV infections and demonstrated a lack of association between condom use and female-reported, male partner circumcision status and HR-and LR-HPV acquisition. Other risks for HR-HPV infection included high socioeconomic status and smoking history, while lifetime use of birth control pills was protective for incident HR-HPV. Our findings demonstrate that HR-HPV is extremely common and underscore the importance of vaccinating young women before they become sexually active.

## Competing interests

The authors declare that they have no competing interests. Vaccine was provided for our trial by GLAXOSMITHKLINE Biologicals, under a clinical trials agreement with NCI. GLAXOSMITHKLINE also provided support for aspects of the trial associated with the regulatory submission needs of the company. NCI and Costa Rican investigators make final editorial decisions on this publication; GLAXOSMITHKLINE has the right to review/comment. The funding agency did not have any involvement in the design of the study; the collection, analysis, and interpretation of the data; the writing of the article; or the decision to submit the article for publication.

## Authors’ contributions

MC: Analyzed data, drafted manuscript. MS: Involved in the conception and design of the study and provided critical revisions of manuscript. ACR: Made substantial contributions to conception and design of study and acquisition of data and provided critical review of the manuscript. SW: Biostatistician, involved in the design of the study and provided critical revisions of manuscript. AH: Made substantial contributions to conception and design of study and acquisition of data and provided critical review of the manuscript. All authors read and approved the final manuscript.

## Pre-publication history

The pre-publication history for this paper can be accessed here:

http://www.biomedcentral.com/1471-2334/13/308/prepub
